# A Systematic Review and Meta-Analysis Assessing the Role of Oral Health as a Risk Factor in Oral Cancer

**DOI:** 10.7759/cureus.39786

**Published:** 2023-05-31

**Authors:** Amit V Mahuli, Vidya Sagar, Amit Kumar, Simpy A Mahuli, Anit Kujur

**Affiliations:** 1 Public Health Dentistry and Preventive Dentistry, Dental College, Rajendra Institute of Medical Sciences, Ranchi, IND; 2 Preventive and Social Medicine, Rajendra Institute of Medical Sciences, Ranchi, IND; 3 Laboratory Medicine, Rajendra institute of Medical Sciences, Ranchi, IND; 4 Dentistry, Rajendra Institute of Medical Sciences, Ranchi, IND

**Keywords:** meta-analysis, oral cancer, periodontal disease, poor oral health, risk factor, tooth loss

## Abstract

Oral squamous cell carcinoma is the leading type of cancer in Southeast Asian countries and many parts of the world. Many factors increase the risk of oral cancer, like tobacco, betel nuts, alcohol consumption, sharp teeth, infections, and other factors. Oral health-related issues have been reported in many studies of oral cancer, but there is a need to understand the role of the same as a risk factor.

The systematic review and meta-analysis were conducted to assess the role of oral health as a risk factor in oral cancer. The population diagnosed with oral cancer (P) of all age groups and both gender, exposure (E) is oral health (includes poor oral hygiene, periodontal disease, and other oral diseases excluding oral potentially malignant disorders (OPMD)), the comparator (C), is patients without oral health issues, outcome (O) is the role of poor oral health as a risk factor for oral cancer.

A systematic review and meta-analysis were conducted. The databases used for the search were PubMed, Cochrane Database, Embase, Scopus, and Google Scholar. The unpublished reports, reviews, and grey literature were considered. Case-control studies were included assessing poor oral health as a risk factor with odds ratio as an effective measure. Newcastle Ottawa Scale for risk of bias in the case-control study was considered.

The study results showed that tooth loss odds ratio (OR)=1.13, CI (0.99-1.26), I^2^ value of 71.7%, Oral hygiene OR=1.29, CI (1.04-1.54), I^2^ value of 19.7% and in periodontal diseases OR=2.14 CI (1.70-2.58), I^2^ value of 75.3% had a higher risk of developing oral cancer. The risk factors for tooth loss and periodontal disease showed moderate heterogenicity and less heterogenicity for oral hygiene.

Poor oral health factors such as periodontal disease, poor oral hygiene, and loss of teeth show higher odds of oral cancer than the control. The periodontal disease shows the highest odds than other factors. These risk factors can be considered for the primordial prevention of oral cancer.

## Introduction and background

With more than 350,000 new cases of oral cancer detected each year, it is a significant public health issue. The incidence of oral cancer varies significantly by geographic location, with Southeast Asia, South Asia, and portions of Europe reporting the highest rates. More than 60% of all occurrences of oral cancer are recorded from Southeast Asian nations, making it the most common type of cancer there, with India accounting for a 9.8 Age-standardized incidence rate in the year 2020 in both genders and all ages of lip and oral cavity cancer. Regarding demographics, men are more likely than women to develop oral cancer, and the risk rises with age [1]. Squamous cell carcinomas, which comprise the bulk of oral malignancies, are linked to the consumption of alcohol, smoking cigarettes, chewing of tobacco, and having the human papillomavirus (HPV) infection [2].

A family history of cancer, poor dental hygiene, persistent mouth inflammation, and occupational exposure to asbestos, nickel, and wood dust are additional risk factors for oral cancer. Oral cancer must be detected early to be successfully treated; hence routine dental exams should include oral cancer screenings. The stage of diagnosis affects the five-year survival rate for oral cancer, with early-stage tumors having a substantially greater survival rate than advanced-stage cancers [3,4].

Alcohol intake reduction, HPV vaccination, and tobacco cessation programs are all effective oral cancer prevention measures. Campaigns for public health education can also significantly impact spreading knowledge about the value of early detection and promoting healthy habits to lower the incidence of oral cancer [5].

Studies on serval populations have revealed a link between oral cancer, periodontal disease, and tooth loss. Because of their increased cellularity, chronic infections produce free radicals that can damage DNA and trigger mutations that culminate in carcinogenesis. Studies have linked oral hygiene (such as brushing) to oral cancer. Only a few research have examined the relationship between the frequency of dental visits and the risk of oral cancer [6-8].

The oral health-related risk factors must be thoroughly investigated, like poor oral health, periodontitis (bleeding gums, mobile teeth), loss of teeth (edentulous state), and other indicators. They can help create awareness and support the primordial prevention of oral cancer. Thus, the systematic review and meta-analysis aimed to assess the role of oral health as a risk factor in oral cancer.

## Review

Methodology

The systematic review and meta-analysis were registered in PROSPERO with the registration number CRD42022311263 dated March 25, 2022 before the start of the study.

PICO Question

The population diagnosed with oral cancer (P) of all age groups and both gender, exposure (I/E) is oral health (includes poor oral hygiene, periodontal disease, and other oral diseases excluding Oral potentially malignant disorders (OPMD)), The comparator (C), is patients without oral health issues, outcome (O), is the role of poor oral health as a risk factor for oral cancer.

Eligibility Criteria and Inclusion

Case-control studies were included in assessing poor oral health as a risk factor. The effect Measure was considered to be an Odds ratio with a Confidence Interval of 95%. Population aged 18 years and above in both genders.

Information Source

The databases used for the search were PubMed, Cochrane Database, Embase, Scopus, and Google Scholar. The unpublished reports, reviews, and grey literature were considered.

Search Strategy

Poor oral health and oral cancer Filters: Abstract, Free full text, Full text, Humans(("poverty"[MeSH Terms] OR "poverty"[All Fields] OR "poor"[All Fields]) AND ("oral health"[MeSH Terms] OR ("oral"[All Fields] AND "health"[All Fields]) OR "oral health"[All Fields]) AND ("mouth neoplasms"[MeSH Terms] OR ("mouth"[All Fields] AND "neoplasms"[All Fields]) OR "mouth neoplasms"[All Fields] OR ("oral"[All Fields] AND "cancer"[All Fields]) OR "oral cancer"[All Fields])) AND ((ffrft[Filter]) AND (fha[Filter]) AND (fft[Filter]) AND (humans[Filter])) with keywords poor oral health, poor oral hygiene, periodontal disease, and oral cancer.

Selection Process

Two reviewers assessed the studies for inclusion. The titles and abstracts of publications were read and categorized first. The texts of the possibly relevant publications were then evaluated, with a final selection of studies being considered for inclusion in the review. The reviewers were educated, and their work was monitored. Responses have been calibrated, and an inter-examiner agreement of more than 80% was expected. A third examiner looked for more relevant material found in the reference lists of the papers and review articles that were included. The fourth and final reviewer was contacted in the event of any discrepancies; he acted as an expert and guide.

Data Extraction

Data were extracted by the authors. Questions about the abstracted data were resolved through discussion by two reviewers working independently among the reviewers. Data from approved research were extracted into a data extraction sheet and then analyzed.

Data Items

Information about the study (author, date, publishing site, total sample size, study type, event rates, odds ratio, confidence interval and the index used to find it). Descriptors of participants (age range, gender, population size, and relevant context). Oral health issues as a risk factor in oral cancer. The periodontal status (bleeding gums, attachment loss), oral hygiene, and tooth loss. Risk of bias was reported using New Castle Ottawa Scale (Table 1) [9].

**Table 1:** Newcastle-Ottawa Quality Assessment Scale case-control studies.

Category Item check Score		
Selection (Maximum four stars)	1. Is the case definition adequate?	1
	2. Representativeness of the cases	1
	3. Selection of controls	1
	4. Definition of controls	1
Comparability (Maximum two stars)	5. Comparability of cases and controls based on the design or analysis	1
		1
Outcome (Maximum three stars)	6. Ascertainment of exposure	1
	7. Same method of ascertainment for cases and controls	1
	8. Non-response rate	1
	Total Score	9

Effect Measure

Odds ratio with a Confidence Interval of 95% was considered.

Synthesis Methods

The descriptive data were collected from the selected studies, and a meta-analysis was carried out to assess the role of poor oral health as a risk factor for oral cancer. The oral health indicators studies were divided into periodontal disease (19 studies), oral hygiene (17 studies) and loss of teeth (24 studies) from a total of 27 full-text studies qualifying to be included in the meta-analysis.

Statistical Plan

The data were analyzed using Stata Version 14; random effects model was used to develop the funnel plot using the Odds ratio, and the confidence interval and degree of heterogeneity were analyzed accordingly. The (I)square value was calculated. Meta-regression analysis was done, and a funnel plot was plotted to check for publication bias. The risk of bias analysis for each study was done using the New Castle Ottawa quality assessment scale for case-control studies, which has three main domains: selection, comparability, Outcome, and a total of nine-point item score.

Results

Study Selection and Characteristics

**Figure 1:**
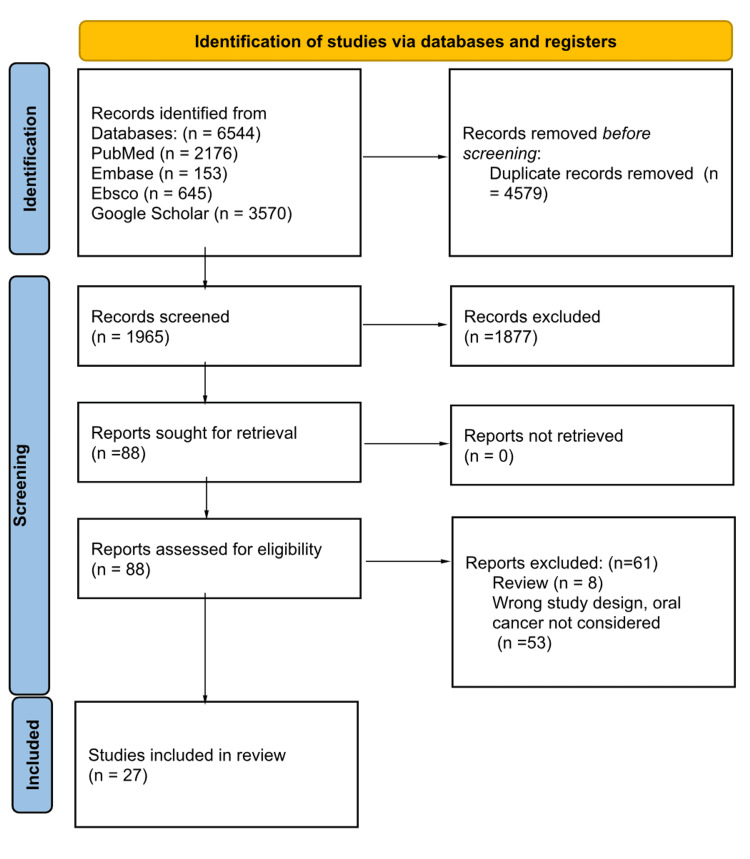
Flow chart showing the selection of the studies for meta-analysis

A total of 1,965 records were included after the search from various databases after the removal of duplicates. Then the articles were screened for appropriateness with the inclusion criteria, mainly studies with keywords like oral cancer and oral health that includes poor oral health, periodontitis (bleeding gums, mobile teeth), loss of teeth (edentulous state) were screened, and 1877 records were excluded. The remaining 88 records were checked for study design (case-control studies), full-text articles, effect measure (odds ratio) and appropriateness of key search terminologies. A total of 27 articles (Table 1) were selected and then divided as articles on periodontal disease (19 articles), articles on oral hygiene (17 studies) and articles on loss of teeth (24 articles) were selected for sub-group analysis (Figure 1).

The risk of bias scores of all articles is mentioned in Table 2 out of a total score of nine [10-36].

**Table 2:** Descriptive characteristics of all the studies involved in the meta-analysis.* *27 articles were extracted, male and female data represented separately for articles and two centers data in one article, total resulting in 30 set of data. M=Male, F=Female, NA=Not applicable

S.No	Author	Year	Study Origin	Mean Age	case sample size	control sample size	Gender	Quality Score	Odds ratio for Tooth loss	Odds ratio for Periodontal disease	odds ratio for oral hygiene
1	Balaram [10]	2002	India	22-85	307	291	Male	9	3.89	2.83	0.96
2	Balaram [10]	2002	India	18-87	278	290	Female	9	7.61	3.35	3.39
3	Bundgaard et al. [11]	1995	Denmark	< 45- >75	161	483	M&F	9	2.4	NA	NA
4	Chang [12]	2013	China	20-80	317	296	M&F	9	2.4	3.11	1.45
5	Divaris [13]	2010	USA	20-80	692	1361	M&F	8	1.05	NA	NA
6	Eliot [14]	2013	USA	18 years and older	148	567	M&F	9	NA	1.07	NA
7	Farquhar et al. [15]	2017	USA	59.4	679	333	M&F	8	0.88	1.24	0.93
8	Guha [16]	2007	Latin american	0-75+	309	1208	M&F	8	0.48	1.94	1.37
9	Guha [16]	2007	Europe	0-75+	146	566	M&F	8	1.2	NA	1.2
10	Gupta [17]	2017	India	57.3	187	240	M&F	7	2.04	3.94	2.16
11	Huang [18]	2015	China	cases 58.02 control57.15	414	870	M&F	9	3.51	NA	NA
12	Kawakita et al. [19]	2017	China	18-85	921	806	M&F	8	1.49	NA	1.13
13	Laprise [20]	2016	India	59.4	306	328	M&F	7	NA	2.28	NA
14	Garrote [21]	2001	CUBA	25-88	200	200	M&F	9	2.74	2.3	1.94
15	Lissowska [22]	2003	Poland	23-80	122	124	M&F	7	9.85	4.33	3.24
16	Marques [23]	2008	Brazil	40-70	168	406	M&F	7	0.7	3	1.5
17	Marshall [24]	1992	USA	18-76	290	290	M&F	7	2.7	NA	NA
18	Mazul et al. [25]	2016	USA	20-80	1396	276	M&F	9	0.85	0.97	NA
19	Moergel [26]	2013	Germany	37-88	178	123	M&F	8	NA	2.4	NA
20	Rosenquist [27]	2005	Sweden	33-87	132	320	M&F	7	3.4	NA	5.3
21	Saira [28]	2019	Pakistan	55 cases, 52.8 controls	276	275	M&F	5	2.56	4.05	2.01
22	Shewale et al. [29]	2021	USA	18-80	114	228	M&F	7	4.55	1.44	2.25
23	Shin [30]	2019	South Korea	63.8 cases, 64.4 controls	146	278	M&F	7	9.99	3.66	NA
24	Talamini [31]	2000	Italy	27-86	132	148	M&F	8	1.4	3.9	1.4
25	Tezal [32]	2009	USA	56.9	100	207	M&F	9	1.02	4.3	NA
26	Tezal [33]	2007	USA	25-87	51	54	M&F	7	0.95	5.23	NA
27	Tezal [34]	2013	USA	58.62 cases, 54.35 controls	399	221	M&F	5	1.57	NA	NA
28	Zheng et al. [35]	1990	China	18-80	248	156	Male	8	3.7	NA	6.9
29	Zheng et al. [35]	1990	China	18-80	248	156	Female	8	8.3	NA	2.5
30	Zuo [36]	2015	China	59	150	167	M&F	6	3.64	NA	NA

Meta-Analysis Findings for Tooth Loss

**Figure 2:**
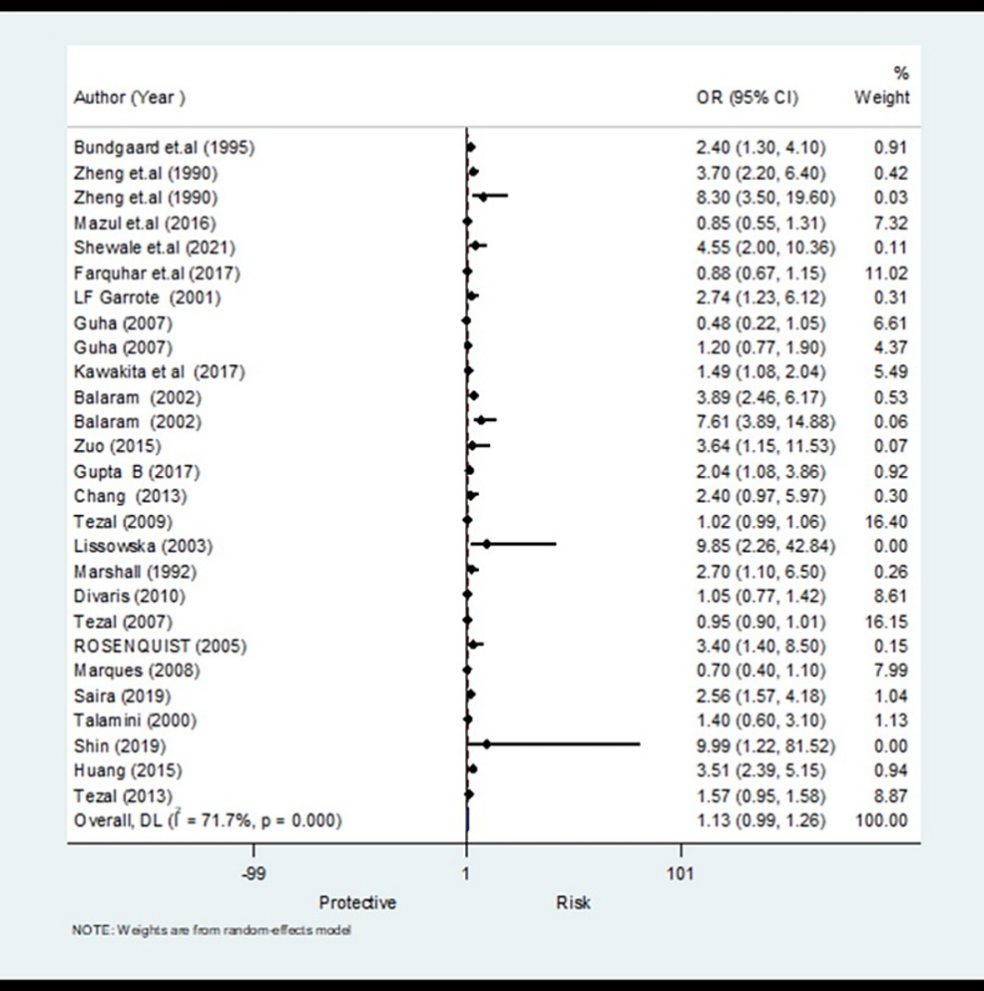
Forest plot showing the meta-analysis results for tooth loss as a risk factor (24 studies) [10-13,15-19,21-25,27-36]

**Figure 3:**
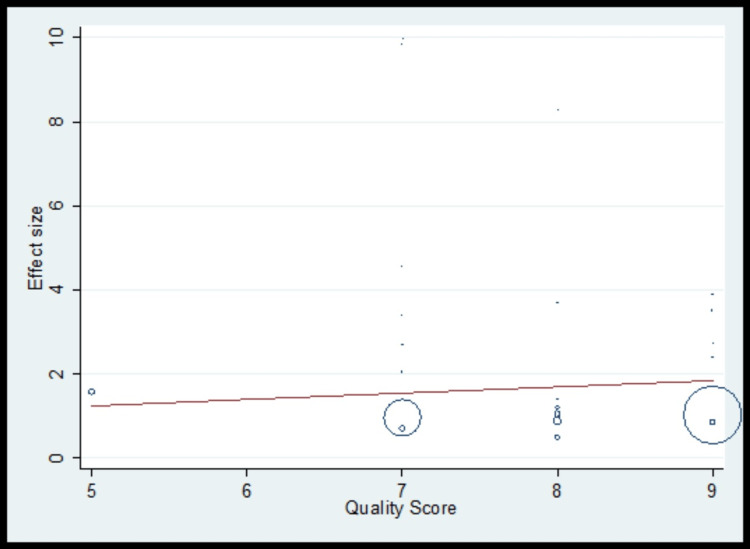
Meta-regression for tooth loss as risk factors

**Figure 4:**
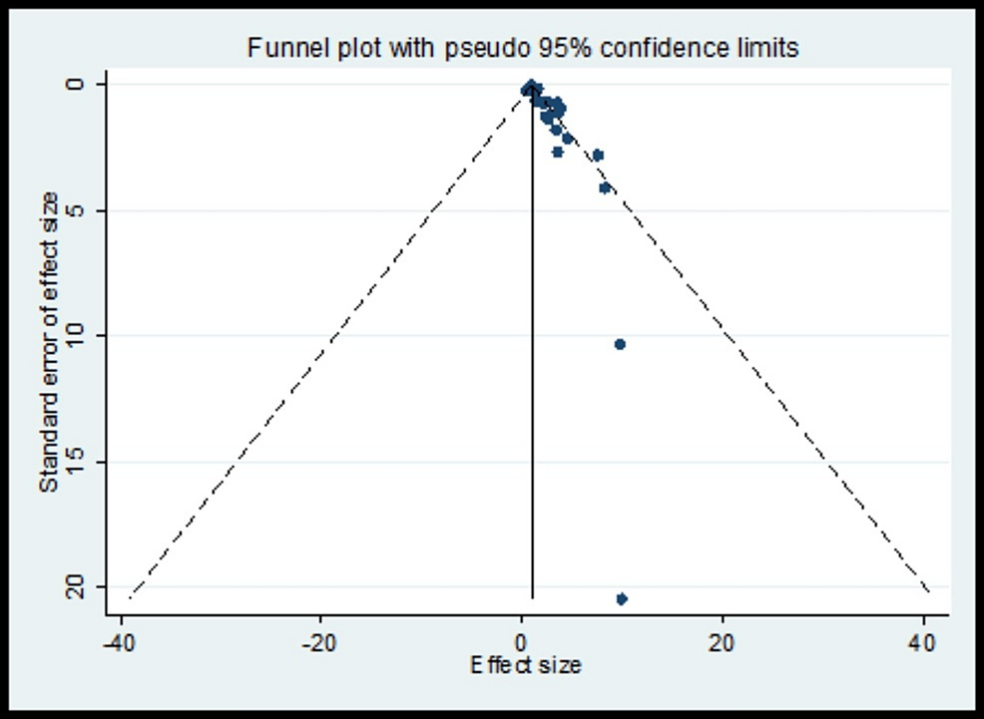
Funnel plot for tooth loss as risk factors for publication bias

The pooled analysis observed that 1.13 times non-significantly higher risk for tooth loss compared to control (OR, 1.13; 95% CI 0.99 to 1.26) with significantly higher heterogeneity (I^2^=71.7%) (Figure 2, Table 3). The meta-regression analysis did not observe the statistically significant heterogeneity due to methodological quality score in the pooled effect size for tooth loss (Figure 3). Funnel plot shows symmetry indicating the absence of publication bias (Figure 4).

**Table 3:** Meta-analysis results for tooth loss as a risk factor.

Results for tooth loss		
Meta-regression	Number of observations	25
REML estimate of between-study variance	tau2	0.7411
% residual variation due to heterogeneity	I-squared_res	71.75%
Proportion of between-study variance explained	Adj R-squared	-15.76%

Meta-Analysis Findings on Oral Hygiene

**Figure 5:**
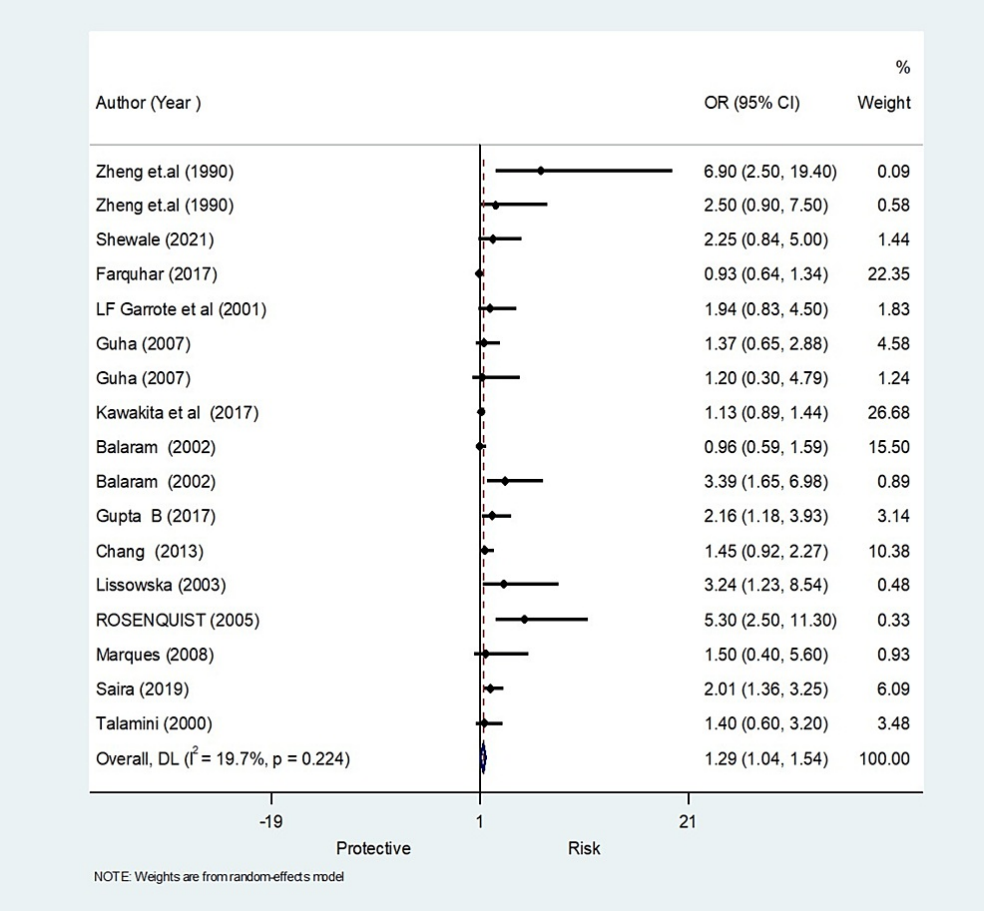
Forest plot showing the meta-analysis results for oral hygiene as a risk factor (17 studies) [10,12,13,15-17,19,21-25,27-33,35,36]

**Figure 6:**
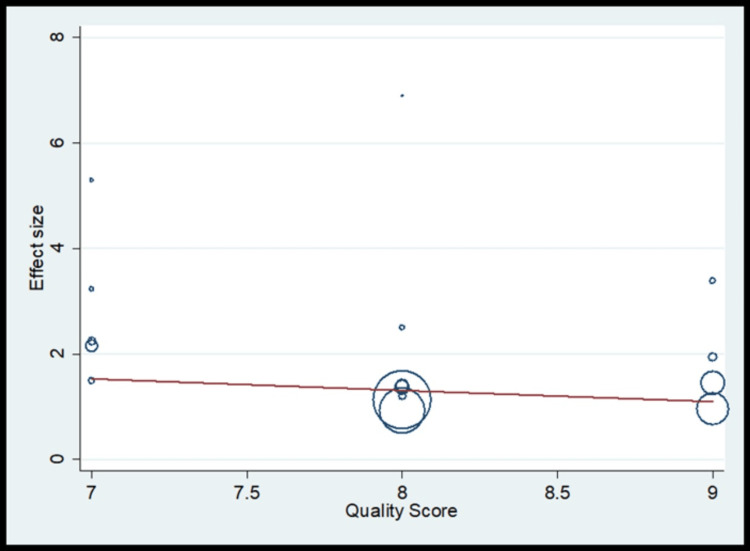
Meta-regression for oral hygiene as risk factors

**Figure 7:**
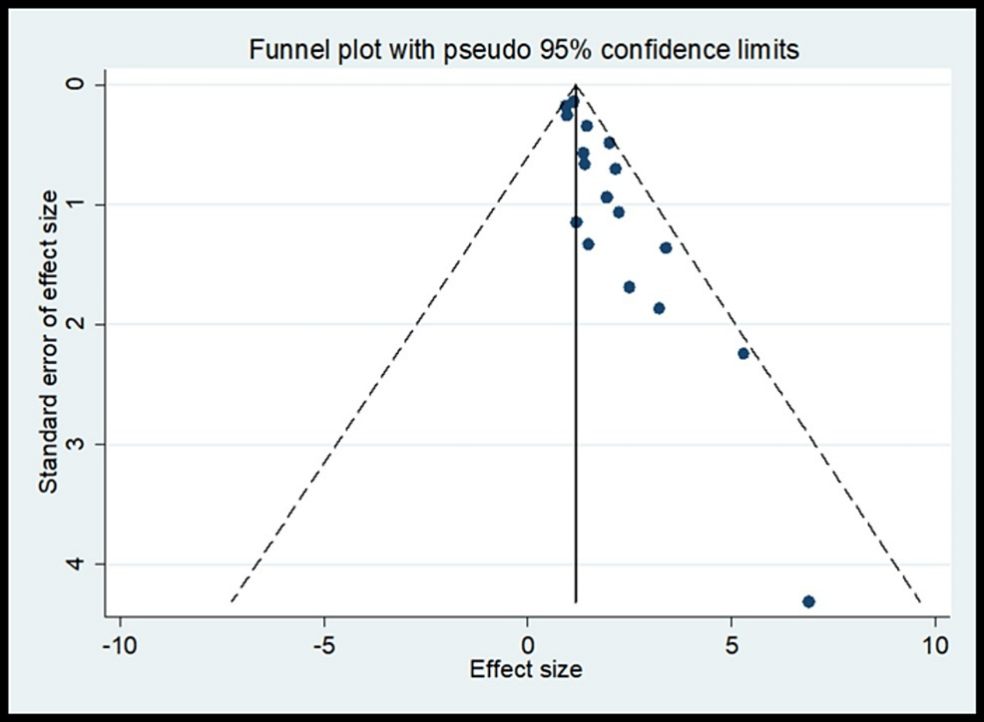
Funnel plot for oral hygiene as risk factors for publication bias

The pooled analysis observed that 1.29 times significantly higher risk for oral hygiene compared to the control (OR, 1.29; 95% CI 1.04 to 1.54) (Figure 5) with homogeneity in finding (I^2^=19.7%) (Table 4). The meta-regression analysis did not observe the statistically significant heterogeneity due to methodological quality score in the pooled effect size for oral hygiene (Figure 6). Funnel plot shows asymmetry indicating the presence of publication bias (Figure 7).

**Table 4:** Meta-analysis results for oral hygiene as a risk factor.

Results for oral hygiene as a risk factor		
Meta-regression	Number of observations	16
REML estimate of between-study variance	tau2	0.05399
% residual variation due to heterogeneity	I-squared_res	15.26%

Meta-Analysis Findings for Periodontal Disease

**Figure 8:**
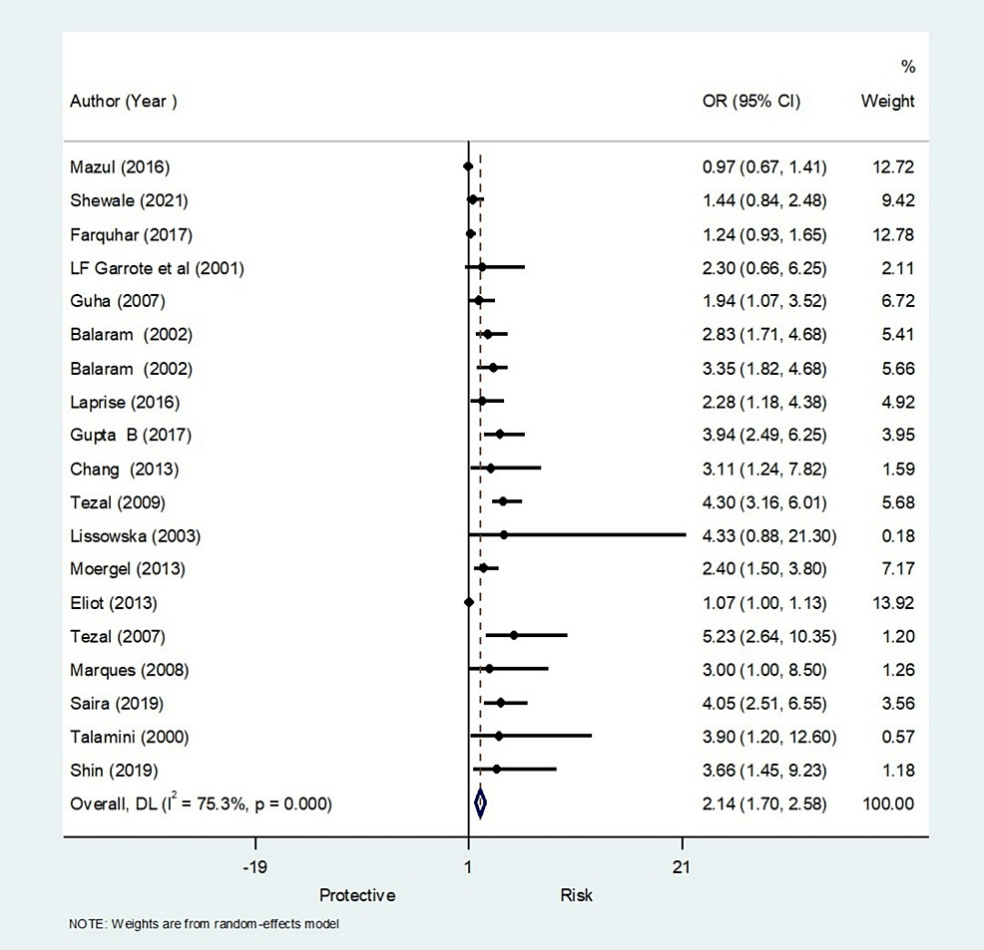
Forest plot showing the meta-analysis results for periodontal disease as a risk factor (19 studies) [10,12,14-17,20-23,25,26,28-33]

**Figure 9:**
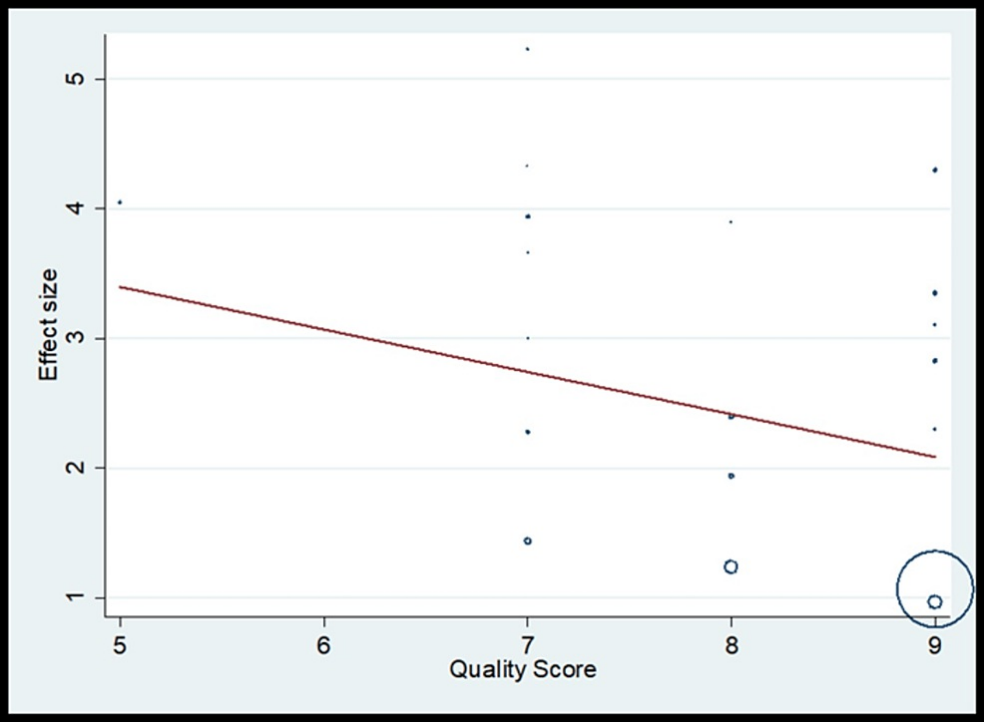
Meta-regression for periodontal disease as risk factors

**Figure 10:**
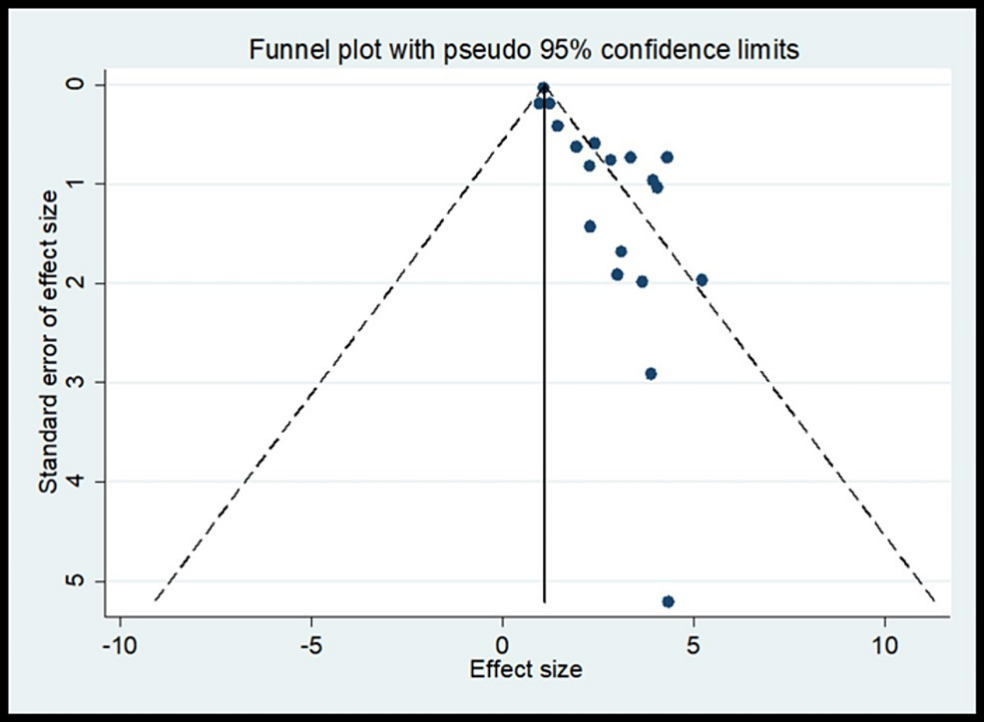
Funnel plot for periodontal disease as risk factors for publication bias

The pooled analysis observed that 2.14 times significantly higher risk for periodontal disease compared to control (OR, 2.14; 95% CI 1.70 to 2.58) (Figure 8) with significant heterogeneity in finding (I^2^=75.3%) (Table 5). The meta-regression analysis did observe the decrease in effect size with an increase in quality for periodontal disease (Figure 9). Funnel plot shows asymmetry indicating the presence of publication bias (Figure 10).

**Table 5:** Meta-analysis results for periodontal disease as a risk factor.

Results for periodontal disease as a risk factor		
Meta-regression	Number of observations	19
REML estimate of between-study variance	tau2	0.9939
% residual variation due to heterogeneity	I-squared_res	68.63%
Proportion of between-study variance explained	Adj R-squared	1.28%

Discussion

The meta-analysis considered the odds ratio as an effect measure. The data extraction was done in detail with the number of cases, controls, case events and control events. The studies included in the analysis didn't show a standardized variable reporting method. Many studies have reported oral health (tooth loss, periodontal disease, oral hygiene) as a risk factor for oral cancer. However, the parameters considered in each of these vary, leading to the heterogenicity of the meta-analysis. Thus, the random-effect model was chosen for the analysis [2,6,7,14].

A total of 24 studies on tooth loss were considered, with 27 entries, as male, female and region-wise data was entered as separate entities from the same studies. Studies reported tooth loss variables in different measurements (missing more than 16, missing more than 20, missing more than five, only five present) leading to moderate heterogenicity of 71.7% (I^2^ value) and an OR of 1.13 and CI of (0.99-1.26), showing higher odds of oral cancer with tooth loss than controls [24,27,31].

The oral hygiene practices showed less heterogenicity of 19.7% (I^2^ value) as brushing habit was recorded uniformly in almost all studies, brushing less than once daily, once daily and twice daily and showed a pooled OR of 1.29, indicating higher odds of oral cancer who less brush (poor oral hygiene) than controls [12,15].­

Likewise, in periodontal disease, measurements considered were gum bleeding, loss of attachment, and bone loss, thus leading to heterogenicity of 75.3% (I^2^ value) [14,20,26,30,32,33]. The presence of periodontal infection shows higher odds (OR 2.14, CI, 1.70-2.58) of oral cancer than the controls, which was statistically significant. The probable reason being reactive oxygen species, reactive nitrogen species, reactive lipids and metabolites, and matrix metalloproteases are also produced by microbes and microbial toxins in host cells and epithelial cells. These substances can potentially damage epithelial cells' DNA and trigger the creation of cytokines, chemokines, growth factors, and other substances that support disrupting the normal control of cell growth and can promote carcinogenesis [7,8].

The oral health risk factors tooth loss, periodontal disease and oral hygiene are the sequel of events that occur from poor oral hygiene (lack of brushing teeth) leading to periodontal disease (gingivitis, periodontitis) and thus to tooth loss. This emphasizes the role of prevention of oral disease and proper oral hygiene, including interdental hygiene, in limiting oral health risk factors in the occurrence of oral cancer [23,26,30]. Many studies have reported poor oral health as a risk factor in nasopharyngeal, pharyngeal and cancer of other head and neck regions. A previous meta-analysis conducted by Shuai in the year 2019 analyzed all methods to describe oral health together and reported a heterogenicity of 85%. Whereas, in the present meta-analysis, tooth loss, oral hygiene and periodontal disease were analyzed individually to get closer to evidence [37].

The meta-analysis highlights periodontal disease as an independent risk factor for oral cancer, followed by poor oral hygiene and tooth loss. However, owing to the moderate heterogenicity and publication bias, there is scope for more studies with a larger sample size. Also, more defined and standardized recording of the variables like, periodontal, oral hygiene and tooth loss need to be followed for more robust analytics. The meta-analysis considered few databases commonly accessible and full text availability of articles were few limitations to be noted. Nevertheless, there is clear evidence that poor oral health can be a risk factor for oral cancer. This indicates good oral hygiene and prevention of oral disease are vital in primordial prevention of oral cancer.

## Conclusions

The periodontal disease shows higher odds of oral cancer when compared to the controls with moderate heterogenicity in the studies considered. The results indicate periodontal disease as a potential risk factor for oral cancer as chronic infection contributes to the occurrence of cancer, followed by oral hygiene with low heterogenicity and tooth loss with moderate heterogenicity; however, there is scope for more multicentric trials or individual studies with a standardized reporting of variables, with larger sample size, event rates and lesser risk of bias in generating more robust evidence. The results indicate that better oral healthcare, oral hygiene practices, and prevention of oral diseases can prevent one of the risk factors leading to oral cancer.

## References

[1] (2023). Globocan 2020 lip and oral cavity factsheet. https://gco.iarc.fr/today/data/factsheets/cancers/1-Lip-oral-cavity-fact-sheet.pdf.

[2] Johnson DE, Burtness B, Leemans CR, Lui VW, Bauman JE, Grandis JR (2020). Head and neck squamous cell carcinoma. Nat Rev Dis Primers.

[3] Hertrampf K, Jürgensen M, Wahl S, Baumann E, Wenz HJ, Wiltfang J, Waldmann A (2022). Early detection of oral cancer: a key role for dentists?. J Cancer Res Clin Oncol.

[4] Silverman Jr S (1988). Early diagnosis of oral cancer. Cancer.

[5] Petersen PE (2009). Oral cancer prevention and control-the approach of the World Health Organization.. Oral Oncol.

[6] Mathur R, Singhavi HR, Malik A, Nair S, Chaturvedi P (2019). Role of poor oral hygiene in causation of oral cancer—a review of literature. Indian J Surg Oncol.

[7] Kavarthapu A, Gurumoorthy K (2021). Linking chronic periodontitis and oral cancer: a review. Oral Oncol.

[8] Karin M, Lawrence T, Nizet V (2006). Innate immunity gone awry: linking microbial infections to chronic inflammation and cancer. Cell.

[9] Wells GA, Shea B, O’Connell D, Peterson J, Welch V, Losos M, Tugwell P (2023). The Newcastle-Ottawa Scale (NOS) for assessing the quality of nonrandomized studies in meta-analyses. http://www.ohri.ca/programs/clinical_epidemiology/oxford.asp.

[10] Balaram P, Sridhar H, Rajkumar T (2002). Oral cancer in southern India: the influence of smoking, drinking, paan-chewing and oral hygiene. Int J Cancer.

[11] Bundgaard T, Wildt J, Frydenberg M, Elbrønd O, Nielsen JE (1995). Case-control study of squamous cell cancer of the oral cavity in Denmark. Cancer Causes Control.

[12] Chang JS, Lo HI, Wong TY (2013). Investigating the association between oral hygiene and head and neck cancer. Oral Oncol.

[13] Divaris K, Olshan AF, Smith J, Bell ME, Weissler MC, Funkhouser WK, Bradshaw PT (2010). Oral health and risk for head and neck squamous cell carcinoma: the Carolina Head and Neck Cancer Study. Cancer Causes Control.

[14] Eliot MN, Michaud DS, Langevin SM, McClean MD, Kelsey KT (2013). Periodontal disease and mouthwash use are risk factors for head and neck squamous cell carcinoma. Cancer Causes Control.

[15] Farquhar DR, Divaris K, Mazul AL, Weissler MC, Zevallos JP, Olshan AF (2017). Poor oral health affects survival in head and neck cancer. Oral Oncol.

[16] Guha N, Boffetta P, Wünsch Filho V (2007). Oral health and risk of squamous cell carcinoma of the head and neck and esophagus: results of two multicentric case-control studies. Am J Epidemiol.

[17] Gupta B, Bray F, Kumar N, Johnson NW (2017). Associations between oral hygiene habits, diet, tobacco and alcohol and risk of oral cancer: a case-control study from India. Cancer Epidemiol.

[18] Huang J, He B, Chen F (2015). Association between oral hygiene, chronic diseases, and oral squamous cell carcinoma (Article in Chinese). Chinese J Preventive Med.

[19] Kawakita D, Lee YA, Li Q (2017). Impact of oral hygiene on head and neck cancer risk in a Chinese population. Head Neck.

[20] Laprise C, Shahul HP, Madathil SA (2016). Periodontal diseases and risk of oral cancer in Southern India: results from the HeNCe Life study. Int J Cancer.

[21] Garrote LF, Herrero R, Reyes RM (2001). Risk factors for cancer of the oral cavity and oro-pharynx in Cuba. Br J Cancer.

[22] Lissowska J, Pilarska A, Pilarski P (2003). Smoking, alcohol, diet, dentition and sexual practices in the epidemiology of oral cancer in Poland. Eur J Cancer Prevention.

[23] Marques LA, Eluf-Neto J, Figueiredo RA (2008). Oral health, hygiene practices and oral cancer. Revista de saude publica.

[24] Marshall JR, Graham S, Haughey BP (1992). Smoking, alcohol, dentition and diet in the epidemiology of oral cancer. Oral Oncol.

[25] Mazul AL, Rodriguez-Ormaza N, Taylor JM (2016). Prognostic significance of non-HPV16 genotypes in oropharyngeal squamous cell carcinoma. Oral Oncol.

[26] Moergel M, Kämmerer P, Kasaj A, Armouti E, Alshihri A, Weyer V, Al-Nawas B (2013). Chronic periodontitis and its possible association with oral squamous cell carcinoma - a retrospective case control study. Head Face Med.

[27] Rosenquist K, Wennerberg J, Schildt EB, Bladström A, Göran Hansson B, Andersson G (2005). Oral status, oral infections and some lifestyle factors as risk factors for oral and oropharyngeal squamous cell carcinoma. A population-based case-control study in southern Sweden. Acta Otolaryngol.

[28] Saira Saira, Ahmed R, Malik S, Khan MF, Khattak MR (2019). Epidemiological and clinical correlates of oral squamous cell carcinoma in patients from north-west Pakistan. J Pak Med Assoc.

[29] Shewale JB, Pickard RK, Xiao W, Jiang B, Gillison ML (2021). Independent association of marijuana use and poor oral hygiene with HPV-negative but not HPV-positive head and neck squamous cell carcinomas. Cancer.

[30] Shin YJ, Choung HW, Lee JH, Rhyu IC, Kim HD (2019). Association of periodontitis with oral cancer: a case-control study. J Dent Res.

[31] Talamini R, Vaccarella S, Barbone F (2000). Oral hygiene, dentition, sexual habits and risk of oral cancer. Br J Cancer.

[32] Tezal M, Sullivan MA, Hyland A (2009). Chronic periodontitis and the incidence of head and neck squamous cell carcinoma. Cancer Epidemiol Biomarkers Prev.

[33] Tezal M, Sullivan MA, Reid ME (2007). Chronic periodontitis and the risk of tongue cancer. Arch Otolaryngol Head Neck Surg.

[34] Tezal M, Scannapieco FA, Wactawski-Wende J (2013). Dental caries and head and neck cancers. JAMA Otolaryngol Head Neck Surg.

[35] Zheng TZ, Boyle P, Hu HF (1990). Dentition, oral hygiene, and risk of oral cancer: a case-control study in Beijing, People's Republic of China. Cancer Causes Control.

[36] Zuo C, Zhu Y, Wang X, Zeng X, Huang C (2015). Tooth loss and risk of oral squamous cell carcinoma in Chinese Han population. Int J Clin Exp Med.

[37] Xu S, Zhang G, Xia C, Tan YH (2019). Associations between poor oral health and risk of squamous cell carcinoma of the head and neck: a meta-analysis of observational studies. J Oral Maxillofac Surg.

