# Correction: A Systematic Review and Meta-Analysis Assessing the Role of Oral Health as a Risk Factor in Oral Cancer

**DOI:** 10.7759/cureus.c344

**Published:** 2025-10-03

**Authors:** Amit V Mahuli, Vidya Sagar, Amit Kumar, Simpy A Mahuli, Anit Kujur

**Affiliations:** 1 Public Health Dentistry and Preventive Dentistry, Dental College, Rajendra Institute of Medical Sciences, Ranchi, IND; 2 Preventive and Social Medicine, Rajendra Institute of Medical Sciences, Ranchi, IND; 3 Laboratory Medicine, Rajendra institute of Medical Sciences, Ranchi, IND; 4 Dentistry, Rajendra Institute of Medical Sciences, Ranchi, IND

This article has been corrected to fix the following typo in the PRISMA diagram: 'Reports excluded' corrected from 63 to 61.

